# How many parents regret having children and how it is linked to their personality and health: Two studies with national samples in Poland

**DOI:** 10.1371/journal.pone.0254163

**Published:** 2021-07-21

**Authors:** Konrad Piotrowski

**Affiliations:** Center for Research on Personality Development, Institute of Psychology, SWPS University, Poznań, Poland; University of Toyama: Toyama Daigaku, JAPAN

## Abstract

Surveys conducted over the last few years on representative samples in the US and Germany suggest that the percentage of parents who regret having children is approximately 17–8%. In none of these studies did the researchers attempt a detailed examination of this group of parents from the perspective of their psychological functioning. In the present article, two studies based on large, national samples (*N* = 1175 and *N* = 1280), one of which was a representative sample of young Poles, are presented. The results obtained show that the percentage of parents who regret parenthood is higher in Poland than in the US or Germany, and that parents who regret having children are characterized by a higher level of adverse childhood experiences, have poorer psychological and somatic health, are more vulnerable to social evaluation, and experience strong parental identity crisis and parental burnout. Regretting parenthood also turns out to be associated with the parent’s financial situation and marital status, and with having children with special needs. The results indicate that regretting becoming a parent is an important social and psychological issue that should become an object of interest for researchers from various disciplines and for social policy authorities.

## Introduction

According to data provided by the Gallup Organization, in 2013 in the US 74% of adults had at least one child, and a further 19% hoped that they would have children in the future; only 5% of the surveyed individuals said that they did not want to have children at all [1]. In a similar survey conducted in the European Union (EU-27) among individuals between the ages of 18 and 40, only 7% of men and 5% of women claimed that they desired a life without children [2]. These data confirm that, despite a general decrease in the fertility rate over the last few decades [3], having children is still an almost universal need of adults. However, becoming a parent, like many other important life decisions, carries a certain risk, as it is a decision whose consequences will accompany the person for the rest of her/his life. Even divorcing one’s partner, breaking off contact with the child and moving to a remote place do not change the person’s awareness of being a parent. Another factor that causes the decision about parenthood to be risky is that one can only find out what it is like to be a parent once one’s child has been born. Taking care of somebody else’s children (e.g. babysitting or taking care of the children of other family members) or working professionally with children (e.g. being a teacher or child psychotherapist) do not provide a full picture of what it is like to have a child. Therefore, as in the case of many other risky decisions, one can presume that some people can also regret the decision to become a parent after it has been made.

Regret is a negative emotion and counterfactual thinking that we experience when we realize that our situation could be better if we had acted differently in the past [4, 5]. As such, regret is conceptualized as an emotional-cognitive phenomenon involved in decision making [6]. Since on some occasions, at least, we make decisions we then regret, the feeling of regret is a natural experience of practically every human being, present in our lives from childhood. In a study conducted by Dijkstra and Barelds [7] on a large sample of Dutch women between the ages of 16 and 81, 65% of the participants said that when they looked back on their lives, they saw things that they would like to have done differently. Although regret is a natural experience, the level of regret has a significant meaning for adjustment. The higher the number of decisions a person regrets, the lower her/his satisfaction with life and the more negative the emotions experienced by her/him [8]. In the cited study by Dijkstra and Barelds [7], the investigated women most frequently regretted decisions made in the sphere of romantic relationships (e.g. getting married too young) and, in turn, they relatively seldom reported that they regretted decisions associated with parenthood; only 10% of the women investigated expressed regret in this domain, connected with, for example, becoming pregnant too early in life (the authors did not provide information about whether any of the women in the investigation regretted having children at all). It has also been observed in other countries that regret over decisions associated with parenthood is infrequent [9, 10]. Perhaps the very fact that regret about decisions connected with becoming a parent is not a common phenomenon is the reason why we know so little about this issue.

The objective of the studies presented here was to fill this important gap in our knowledge about contemporary parents, and to answer two questions: *What is the percentage of parents who regret that they decided to have a child*? and *What are the demographic*, *social*, *and psychological characteristics of such parents*? Both of these research questions seem very important, because the review of the scientific literature clearly indicates that we have only scarce knowledge about these issues. At the same time, knowledge about how many parents regret making the decision about parenthood, and what causes this regret, may be significant, not only for representatives of many branches of science, including psychology, psychiatry, sociology, and public health, but also for the social policy of the many countries that treat the quality of life of the family and the child as one of their priorities [11]. The research presented here is the first to carry out a thorough investigation of the issue of regretting parenthood in a representative sample of parents, while simultaneously taking into consideration various socio-psychological conditionings of this phenomenon.

### How many parents regret having a child?

Although a situation in which a parent regrets having a child can have serious social, emotional, and health-related consequences, researchers (not only psychologists, but also researchers from other branches of the social sciences) have so far failed to explore this issue. As a result, our knowledge about the frequency of regretting parenthood can be derived mainly from the small number of survey studies conducted by non-governmental institutions, which provide only limited information. In a survey conducted by the Gallup Organization [1], Americans above the age of 45 who had children were asked how many children they would like to have had if they could *do it once again*. One of the possible answers was ‘0’, which turned out to be the answer selected by 7% of the surveyed individuals. In this survey, though, the authors did not ask directly whether the parent would again decide to have or not to have children, which is why a certain dose of carefulness is required in interpreting these results. Additionally, the study conducted by the Gallup Organization provided information neither about the social structure of the surveyed group nor about its psychological or sociological characteristics. In a similar survey, conducted in 2016 on a representative sample of Germans with children [12, 13], the authors asked the participants to react to the following statement: *If I could choose today once again*, *I would not want to have children*. It turned out that 8% of the German parents in the investigation said that they *fully agree with this*, and another 11% claimed that they *rather agree with this*. What distinguished the group of parents who either fully or somewhat agreed with this statement was a belief that being a parent had limited their ability to develop further personally and professionally, and that they had had to sacrifice too much for their families. These individuals also experienced lower satisfaction from parenthood than the parents who would once again decide to have children. Regretting parenthood was also more frequent among individuals with a lower income (below 1500 euros per household). The German study suggests that one of the reasons why some people regret parenthood is a belief that because one is a parent one cannot have the life that one would like. The two studies mentioned above are, according to the best of the author’s knowledge, the only surveys that provide us with a notion of the percentage of parents from different countries who regret choosing the path of being a parent. It is worth paying attention to the fact that the American and the German studies suggest a comparable proportion of around 7–8%. On the other hand, the studies were conducted on different groups (individuals above the age of 45 in the US, and individuals above the age of 18 in Germany). Also, these studies do not give us an opportunity to find out more about the groups of parents. Luckily, in the last few years several studies have been conducted, albeit on small samples, that have shed some light on the functioning of parents who regret having children.

### What are the characteristics of parents who regret having a child?

Donath [14], in her groundbreaking work dedicated to regretting parenthood, has suggested that one of the more important causes of this phenomenon is strong, cultural pressure on women, under which they feel obliged to have a child if they are to be fully accepted by society. For this reason, many women decide to have a child even when they are not fully convinced that this is the right decision. An analysis of parental internet fora on websites such as Mumsnet and Reddit [15, 16] suggests, in fact, that mothers who regret having a child often think that they do not fit this role, that it disables them from self-realization, and that it leads to them losing their true identity. Additionally, they have a tendency to return in their minds to the times “before the child”, which are described as times of freedom and self-fulfillment. Studies on parental identity conducted by Piotrowski [17] also suggest that regretting parenthood can be a consequence of low identification with the parental role and of a conviction that not having a child would give a better fit with one’s life. Although Piotrowski did not ask parents directly whether they regretted having a child, his studies suggest that, among parents who regret having children, such phenomena as negative emotionality and poor mental health may be observed. This accords with observations indicating that strong and long-lasting experiences of regret are associated with a low quality of life [8]. In turn, East, Chien, and Barber [18] have demonstrated that the relationship between regretting parenthood and mental health can have a reciprocal character. The authors conducted a longitudinal study on 100 Mexican American female adolescents. At the beginning of the study, the adolescents were pregnant (third trimester) with their first child, and they were then examined when their children were 6 months old and 12 months old. The authors observed that regretting having a child was positively associated with levels of depression, anxiety, and parental stress, but also with a harsher and more rejecting attitude towards the child. Between parental stress and regretting parenthood, the authors also observed a reciprocal relation, which suggests that they can mutually reinforce and stimulate one another. East, Chien, and Barber [18] explain that having a child during adolescence is an enormous challenge, and that high stress can hamper everyday functioning in the role of parent and partner, leading to a conviction that it would be better not to have the child, which, in further steps, is conducive to experiencing higher stress and increased mental problems.

These few studies on regretting parenthood that have been carried out so far enable us to make two main observations. First, regretting parenthood may pertain to as many as every one in ten or eleven parents, which on the scale of countries such as the United States (over 300 million inhabitants) or Germany (over 80 million inhabitants) translates into millions of parents, making it an issue of great social significance. Secondly, regretting parenthood can be strongly personality-conditioned [17] and can further increase psychopathology among parents and destructive attitudes towards their children [18]. Because the studies on this issue that have been conducted so far have concentrated either on determining the prevalence of parental regret in the population [1, 12] or on determining the correlates of this phenomenon in relatively small samples [14–16, 18], it was decided to integrate these two research strategies. The studies presented here, conducted on large, nation-wide, Polish samples, were carried out in order to, first of all, determine the frequency of regretting parenthood among parents in a large, European, EU member country, and, secondly, to study the characteristics of this group of people from the point of view of demographic, social, and psychological factors. To the best of the author’s knowledge, this is the first study in which such an approach to the analysis of regretting parenthood has been followed. As research on such a rarely discussed yet socially important topic, which can significantly influence public opinion, needs to be verified using different samples, it was decided to conduct two separate studies with different, randomly selected participants and compare their results to increase the reliability of the results. Their presentation in a single article was, in turn, dictated by the desire to offer the reader as a comprehensive picture of this almost unknown phenomenon as possible.

### Research problem and hypotheses

In order to answer the research questions, two cross-sectional studies were conducted. Both of them focused on relatively young parents (in Study 1, the participants were between the ages of 18 and 40, whereas in Study 2, they were between 18 and 30 years old), or, in other words, on individuals who because of their young age were still actively engaged in the realization of the parental role (the chance at this age of having children who are already independent is rather small). On the basis of the surveys conducted in the US and in Germany, it was assumed that the percentage of parents regretting having a child could be around 7–8% (H1). It was predicted that regretting becoming a parent could, to a certain extent, be associated with demographic factors (H2), and, according to the results obtained in the German study [12], regretting parenthood could occur more frequently among individuals who experience substantial financial difficulties. On the basis of the results obtained by East, Chien, and Barber [18], it was also hypothesized that parents who regretted having a child would be characterized by greater mental and somatic health difficulties (e.g. depression, anxiety, vegetative symptoms) and a lower quality of life (H3).

## Study 1

As mentioned above, the available studies suggest that parents who regret having a child are characterized by a lower quality of life and are more prone to psychopathology. In Study 1, it was decided to verify this relationship, which so far has not been studied, for a representative sample, and to broaden our knowledge by looking at additional aspects of the functioning of this group of parents. It was assumed that chronic stress and the difficulties associated with regretting parenthood would translate into higher levels of vegetative symptoms (e.g. symptoms in the digestive system, fast heart rate, and high blood pressure) or recurring aches in various parts of the body [19]. In Study 1, it was predicted that such difficulties would be experienced more often by parents who regretted having children.

Another issue analyzed in Study 1 was the early conditionings of regretting parenthood. For this purpose, levels of traumatic childhood experiences among parents who regretted parenthood and among those who did not were evaluated. Because adverse childhood experiences find their reflection in lower adaptation of individuals in the periods of adolescence and adulthood, and in their poorer mental and physical health [20, 21], it was assumed that the risk of regretting parenthood would be higher in individuals who had endured this kind of trauma.

### Participants and procedure

The study was reviewed and approved by the Departmental Ethics Committee, SWPS University of Social Sciences and Humanities, Poznań, Poland (decision number 190110). The participants provided their written informed consent to participate in this study.

Study 1 was conducted with the participation of a representative sample of Poles in the period of emerging and early adulthood (aged 18–40). The sample was gathered randomly, from a nationwide research panel that rewards research participants. The recruitment for the study was carried out in such a way as to reflect best the distribution of age, education, and place of residence in the population. Although the frequency of these variables observed in the final sample differed slightly from their distribution in the population, the differences were minimal, being at most several percentage points. As such, the studied sample can be considered representative of the population of Polish parents aged 18–40. The study was carried out online with the use of the internal research platform being the functionality of the research panel. The investigated individuals who were drawn were sent a link that enabled them to participate in the study described as a research project on parenting’s good and bad sides. An invitation to participate had been randomly sent to those research panel members who fulfilled the criteria of age (from 18 to 40) and were registered in the research panel as having at least one child (these two criteria were additionally verified during the study with questions about age, having children, number of children, etc.). Because it was mandatory to provide answers to all the questions, there were no missing data in the database. The participants were informed about the aim of the study, and gave their written consent to participate. The study was approved by the Departmental Ethics Committee of the author’s institution.

A total of 1175 individuals between the ages of 18 and 40 (*M =* 31.63, *SD* = 6.30) took part in the study. Women constituted 57.4% of the sample; 28% of the participants lived in villages, whereas 72% lived in town and cities of different sizes; 30% of the participants had higher education or were studying at the time of the survey, 22% had secondary education, and 47% had primary education, basic vocational education or the equivalent. The percentage of the participants who were married was 62.6%, 29.9% of them were in informal relationships, and 7.5% were single at the time of the study; 31.7% of the investigated individuals claimed that they did not have any financial problems, 58.4% said they had minor financial problems, but considered their situation to be average, and 10% claimed they had substantial financial problems; 49.5% of the participants had one child, 35.7% had two children, 10.6% had three children, and the remaining 4.2% had more than three children. The average age of the participants’ children was 6.47 years (*SD* = 5.05).

### Measures

#### Regretting parenthood

To verify whether the participants regretted having a child/children, they were asked the following single question: *If you could travel back in time and once again make the decision*, *would you once again decide to become a parent*? Since the aim of the study was to acquire an unequivocal opinion from the parents, they could choose only one of two options: (1) *No*, *I would choose a life without children*, or (2) *Yes*, *I would choose to have children*.

#### Depressive, anxiety, and vegetative symptoms

In order to evaluate the participants’ mental and somatic health, the Symptom Checklist-27-plus Questionnaire was used (SCL-27-plus; [19]; Polish adaptation [22]). This measure consists of five subscales designed to evaluate the current existence of the following symptoms, respectively: 1. Depressive symptoms (5 items, e.g. *hopelessness*, *feeling blank inside;* Cronbach’s alpha: .93), 2. Vegetative symptoms (5 items, e.g. *nausea*, *heart pounding;* Cronbach’s alpha: .85), 3. Agoraphobic symptoms (5 items, e.g. *fear of leaving the house alone*, *becoming afraid in crowds;* Cronbach’s alpha: .91), 4. Social phobia symptoms (5 items, e.g. *fear of saying something embarrassing*, *feeling of being unwanted;* Cronbach’s alpha: .90) and 5. Pain (6 items, e.g. *headache*, *backaches;* Cronbach’s alpha: .82). The investigated individuals evaluated the frequency of experiencing the particular symptoms with the use of a Likert scale, ranging from 1-*never* to 5-*very often*. This scale also includes an indicator of the existence of symptoms of depression during the person’s lifetime (Cronbach’s alpha: .88), assessed on a scale of 0–1. The scale also contains questions about attempting suicide. Still, these were not used in the reported study as suicidality was not a focus of the project, and it was decided to make the survey as short as possible so as not to burden the participants.

#### Traumatic experiences in childhood

A short form of the Childhood Trauma Questionnaire (CTQ-SF; [23]; Polish adaptation [24]) was used to evaluate the level of neglect and abuse from family members in childhood. The measure consists of 28 items, 25 of which create five subscales: Emotional abuse (5 items; e.g. *People in my family called me things like “stupid”*, *“lazy”*, *or “ugly”;* Cronbach’s alpha: .88), Physical abuse (5 items; e.g. *People in my family hit me so hard that it left me with bruises or marks*; Cronbach’s alpha: .93), Sexual abuse (5 items; e.g. *Someone threatened to hurt me or tell lies about me unless I did something sexual with them*; Cronbach’s alpha: .96), Emotional neglect (5 items; e.g. *I felt loved*, reverse coded; Cronbach’s alpha: .90), and Physical neglect (5 items; e.g. *My parents were too drunk or high to take care of the family*; Cronbach’s alpha: .77). All items are assessed on a 5-point Likert scale, ranging from 1-*Never true* to 5-*Very often true*. The three additional items create a control scale, indicating a possibility of underreporting of maltreatment; these were, however, omitted due to time constraints and the desire not to burden the subjects unnecessarily.

### Analytical strategy

The descriptive statistics, mean differences and correlations between the quantitative variables were checked in the first step. Next, the distribution of answers to the question about regretting parenthood was verified in the whole sample (H1). The next step was the evaluation of relationships between regretting parenthood and demographic factors using the *Chi-*Square test (H2). The factors were gender, marital status (married, informal relationship, single), and financial situation (lack of financial problems, minor financial problems, substantial financial problems). Additionally, also a place of residence (village, cities of different sizes) and education (primary/vocational, secondary, higher or currently studying) were included. In the last step, differences between parents who regretted having children and those who did not regret becoming parents regarding mental and somatic symptoms and traumatic experiences in childhood (H3) were analyzed using multivariate analysis of variance (MANOVA).

### Results

#### Descriptive statistics, mean differences, and correlations between variables

The descriptive statistics and the differences between females and males, participants with different relationship statuses, and those with different financial situations are presented in Table 1. The results showed that women scored significantly higher on the dimensions indicating poor mental (depressive symptoms, social phobia, agoraphobia) and somatic (pain and vegetative symptoms) health. At the same time, men experienced more intense physical abuse and physical neglect as children. Further analyses showed that those parents who were married during the study or had not had serious financial problems scored lower on health problems and childhood trauma. These observations are in accord with other studies in which the links between gender and marital and financial status and parental adjustment were analyzed [25, 26].

**Table 1:** Descriptive statistics and mean differences in a study sample (Study 1).

	Range	All sample (*N* = 1175)	Gender			Marital status				Financial situation			
			Female	Male		Married	Informal	Single		No financial problems	minor financial problems	substantial financial problems	
			(*n* = 674)	(*n* = 501)		(*n* = 736)	(*n* = 351)	(*n* = 88)					
										(*n* = 372)	(*n* = 686)	(*n* = 117)	
		*M (SD)*	*M (SD)*	*M (SD)*	*F / p*	*M (SD)*	*M (SD)*	*M (SD)*	*F / p*	*M (SD)*	*M (SD)*	*M (SD)*	*F / p*
Depressive symptoms (SCL)	1–5	2.04 (.97)	2.11 (.99)	1.93 (.93)	11.08**	1.89^a^ (.89)	2.22^b^ (1.01)	2.52^c^ (1.14)	26.44***	1.82^a^ (.89)	2.06^b^ (.95)	2.59^c^ (1.10)	29.63***
Lifetime depressive symptoms (SCL)	0–1	.38 (.40)	.42 (.40)	.32 (.38)	16.30***	.32^a^ (.38)	.46^b^ (.40)	.52^b^ (.39)	21.30***	.32^a^ (.38)	.38^a^ (.39)	.56^b^ (.40)	17.19***
Vegetative symptoms (SCL)	1–5	2.26 (.77)	2.31 (.78)	2.19 (.77)	6.58*	2.19^a^ (.78)	2.35^a,b^ (.74)	2.47^b^ (.81)	8.86***	2.15^a^ (.78)	2.29^a,b^ (.76)	2.44^b^ (.81)	7.59**
Agoraphobia symptoms (SCL)	1–5	1.83 (.95)	1.89 (.96)	1.76 (.93)	5.55*	1.75^a^ (.93)	1.98^b^ (.93)	2.06^b^ (1.01)	9.92***	1.70^a^ (.91)	1.89^a,b^ (.95)	2.01^b^ (1.00)	6.81 **
Social phobia symptoms (SCL)	1–5	2.29 (.98)	2.40 (1.01)	2.15 (.91)	20.02***	2.19^a^ (.96)	2.43^b^ (.97)	2.59^b^ (1.01)	11.87***	2.16^a^ (.98)	2.31^a^ (.96)	2.62^b^ (1.02)	10.15***
Symptoms of pain (SCL)	1–5	2.63 (.70)	2.68 (.70)	2.57 (.69)	7.35**	2.59 (.69)	2.69 (.69)	2.69 (.77)	*ns*	2.49^a^ (.70)	2.68^b^ (.68)	2.77^b^ (.74)	11.54***
Emotional abuse (CTQ)	1–5	1.86 (.90)	1.89 (.93)	1.81 (.86)	*ns*	1.74^a^ (.82)	2.05^b^ (.99)	2.08^b^ (1.00)	17.91***	1.75^a^ (.87)	1.88^a,b^ (.89)	2.06^b^ (1.03)	6.18**
Physical abuse (CTQ)	1–5	1.54 (.83)	1.48 (.81)	1.61 (.85)	6.47*	1.48^a^ (.78)	1.64^b^ (.91)	1.65^a,b^ (.91)	5.39**	1.45^a^ (.79)	1.57^a,b^ (.84)	1.64^b^ (.89)	3.29*
Sexual abuse (CTQ)	1–5	1.33 (.77)	1.30 (.74)	1.36 (.81)	*ns*	1.28^a^ (.70)	1.40^b^ (.85)	1.45^a,b^ (.95)	4.24*	1.27^a^ (.72)	1.34^a,b^ (.77)	1.45^b^ (.92)	*ns*
Emotional neglect (CTQ)	1–5	2.36 (.96)	2.35 (1.01)	2.38 (.89)	*ns*	2.28^a^ (.94)	2.48^a,b^ (.97)	2.61^b^ (1.02)	8.51***	2.22^a^ (.91)	2.41^a,b^ (.96)	2.54^b^ (1.06)	7.20**
Physical neglect (CTQ)	1–5	1.89 (.75)	1.83 (.77)	1.97 (.73)	9.72**	1.83^a^ (.74)	1.98^a,b^ (.77)	2.04^b^ (.77)	6.81**	1.74^a^ (.71)	1.94^b^ (.75)	2.09^b^ (.80)	13.71***

* *p* < .05

** *p* < .01

*** *p* < .001, *ns*—not significant; post-hoc test Tukey HSD (groups with different index differed significantly).

Note: SCL: the Symptom Checklist-27-plus Questionnaire; CTQ: the Childhood Trauma Questionnaire.

The correlations between the quantitative variables are presented in Table 2. The indicators of traumatic experiences in childhood (emotional, physical, and sexual abuse, and emotional and physical neglect) turned out to be positively and, in most cases, moderately strongly associated with all of the indicators of mental and somatic health issues. These results remain in accord with reports about the negative influence of childhood trauma on functioning in adulthood [20, 21]. It was also verified whether the studied constructs correlate with the parent’s age, the mean age of the children/age of the only child, and the number of children. As shown in Table 2, children’s age and number were only marginally correlated with some of the studied constructs. However, parents’ age turned out to be associated with most of the other variables. Younger parents experienced more depressive, anxiety, and vegetative symptoms, which is in line with studies on the prevalence of psychological issues in different age groups [27]; at the same time, younger parents scored lower on two of the childhood trauma indicators (emotional abuse and neglect) which might be the effect of changes in parenting practices over time.

**Table 2:** Correlations between study variables (Study 1).

	2	3	4	5	6	7	8	9	10	11	12	13	14
1. Depressive symptoms (SCL)	.60***	.56***	.57***	.65***	.45***	.53***	.41***	.37***	.30***	.39***	-.16***	-.03	-.01
2. Lifetime depressive symptoms (SCL)	-	.42***	.43***	.48***	.34***	.42***	.27***	.22***	.25***	.25***	-.13***	-.03	.01
3. Vegetative symptoms (SCL)		-	.68***	.65***	.73***	.45***	.42***	.36***	.15***	.32***	-.10**	.02	.04
4. Agoraphobia symptoms (SCL)			-	.73***	.48***	.48***	.43***	.44***	.21***	.38***	-.16***	-.05	.01
5. Social phobia symptoms (SCL)				-	.50***	.52***	.37***	.32***	.29***	.34***	-.19***	-.10***	.01
6. Symptoms of pain (SCL)					-	.37***	.35***	.27***	.11***	.23***	.01	.09**	.09*
7. Emotional abuse (CTQ)						-	.77***	.61***	.56***	.67***	-.15***	-.06	.05
8. Physical abuse (CTQ)							-	.71***	.38***	.68***	-.01	.04	.10**
9. Sexual abuse (CTQ)								-	.21***	.56***	-.01	.01	.07*
10. Emotional neglect (CTQ)									-	.62***	-.08**	-.01	.03
11. Physical neglect (CTQ)										-	-.06	.03	.13***
12. Age of the parent											-	.68***	.28***
13. Mean age of the children (age of the only child)												-	.26***
14. Number of children													-

* *p* < .05

** *p* < .01

*** *p* < .001.

Note: SCL: the Symptom Checklist-27-plus Questionnaire; CTQ: the Childhood Trauma Questionnaire.

#### Regretting parenthood in the study sample

The analysis of the answers obtained to the question about regretting the decision about becoming a parent indicated (Table 3) that in the population of the Polish parents between the ages of 18 and 40, 13.6% of the parents claimed that if they could once again make the decision, they would choose a life without children.

**Table 3:** Percentage of Polish parents aged 18–40 who regretted having a child (Study 1).

If you could travel back in time and once again make the decision, would you once again decide to become a parent?	No, I would choose a life without children	Yes, I would choose to have children
	*n* = 160	*n* = 1015
	13.6%	86.4%

#### Social and demographic factors in parents regretting parenthood

Table 4 presents the relationships between regretting parenthood and socio-demographic factors: gender, marital situation, and parents’ financial situation. The results of the *Chi-*square analysis show that the percentage of parents who regretted having a child was comparable among women and men. It was observed, however, that the percentage of the parents who regretted having a child was markedly higher among the single parents (those who did not have a partner at the time of the study; 27.3% of them regretted parenthood), whereas among the parents who had remained in an informal relationship this percentage was 18.2%, and among the parents who were married it was 9.8%, *Chi*^2^ (2) = 29.51, *p* < .001, Cramer’s *V* = .16. Another significant correlate of regretting having a child turned out to be financial situation, although this relationship was weaker than the previous one, *Chi*^2^ (2) = 7.08, *p* < .05, Cramer’s *V* = .08. Among the parents who claimed that their financial situation was bad or rather bad, the percentage who regretted parenthood was 21.4%, whereas among the individuals who only sometimes experienced minor financial problems this percentage was 12.2%, and among the parents who did not have financial problems it was 13.7%.

**Table 4:** Regretting parenthood and gender, marital status, and financial situation (Study 1).

	If you could travel back in time and once again make the decision, would you once again decide to become a parent?		
	No, I would choose a life without children	Yes, I would choose to have children	*Chi*^2^
			*p*
	*n* = 160	*n* = 1015	
Gender			
Female (*n* = 674)	13.1%	86.9%	*ns*
Male (*n* = 501)	14.4%	85.6%	
Marital status			
Married (*n* = 736)	9.8%	90.2%	*X*^2^ = 29.51
Informal relationships (*n* = 351)	18.2%	81.8%	*p* < .001
Single (*n* = 88)	27.3%	72.7%	Cramer’s *V* = .16
Financial situation			
No financial problems (*n* = 372)	13.7%	86.3%	*X*^2^ = 7.08
Minor problems (*n* = 686)	12.2%	87.8%	*p* < .05
Bad financial situation (*n* = 117)	21.4%	78.6%	Cramer’s *V* = .08

Additionally, it turned out that among the individuals living in villages and those living in cities of different sizes, and among the individuals with varying levels of education, the percentage of parents who regretted having a child was similar. There were also no differences in respect of the age of the participants or their children’s ages between the two groups.

#### Psychological functioning and health in parents regretting parenthood

Table 5 presents the differences between the two groups of parents regarding the dimensions of the SCL-27 and the CTQ. The MANOVA revealed a significant multivariate effect, Wilks’ *λ* = .91, *F* (11, 1163) = 9.96, *p* < .001, *η*^2^ = .09. Significant differences between the groups could be observed in the case of every one of the analyzed variables. The parents who regretted having children were distinguished by higher levels of mental, vegetative and pain-related problems, and they had also experienced to a greater degree different forms of violence and neglect in childhood. Analyzing the effect size coefficient, we can see that the greatest differences between the groups pertained to the current levels of depressive symptoms (depressive symptoms, SCL) and emotional and physical abuse in childhood (CTQ).

**Table 5:** Mean differences between the parents regretting and not regretting parenthood in Study 1.

	No, I would choose a life without children	Yes, I would choose to have children		
	*M (SD)*	*M (SD)*	*F*	eta^2^
Depressive symptoms (SCL)	2.62 (1.07)	1.94 (.92)	69.31***	.06
Lifetime depressive symptoms (SCL)	.56 (.40)	.35 (.39)	40.57***	.03
Vegetative symptoms (SCL)	2.61 (.83)	2.20 (.75)	38.97***	.03
Agoraphobia symptoms (SCL)	2.31 (1.10)	1.76 (.90)	48.35***	.04
Social phobia symptoms (SCL)	2.78 (1.09)	2.22 (.94)	47.65***	.04
Symptoms of pain (SCL)	2.87 (.73)	2.59 (.69)	22.85***	.02
Emotional abuse (CTQ)	2.44 (1.02)	1.77 (.84)	83.19***	.07
Physical abuse (CTQ)	2.01 (1.08)	1.46 (.76)	62.57***	.05
Sexual abuse (CTQ)	1.73 (1.09)	1.27 (.68)	52.11***	.04
Emotional neglect (CTQ)	2.73 (.95)	2.30 (.95)	28.15***	.02
Physical neglect (CTQ)	2.25 (.78)	1.84 (.73)	43.63***	.04

* *p* < .05, ** *p* < .01, *** *p* < .001.

Note: SCL: the Symptom Checklist-27-plus Questionnaire; CTQ: the Childhood Trauma Questionnaire.

## Study 2

In Study 2, the focus was on verifying the findings of Study 1 with a different sample and on acquiring further information about the group of parents who regretted having a child. According to Donath [14], regretting parenthood is caused, at least to some extent, by the cultural pressure that young people experience. For this reason, one can suppose those personality traits associated with the greater sensibility to the evaluation and opinions of other people are at higher levels in those parents who undertake this role, not because of their internal needs but because of external motivations. An external locus of control and a fear of negative evaluation from others are characteristic traits of maladaptive aspects of perfectionism [28]. For this reason, it was assumed that these aspects of perfectionism, which are associated with a fear of negative evaluation, have higher levels among parents who regret parenthood. It has been hypothesized that these may be the individuals who often try to fulfill expectations and find it more difficult to resist the cultural, social, and familial pressure to have children.

In Study 2, the suggestions formulated by Piotrowski [17], that regretting parenthood could be the consequence of an inability to form a stable parental identity and of parental burnout, were also verified. Difficulties in forming parental identity manifest themselves mainly in low identity commitment, low identification with the role of a parent, and the lack of active exploration of the parental domain [29, 30]. In turn, strong parental burnout [31] manifests itself in the sense of being tired with parenthood, an increasing conviction that one is a bad parent, and a withdrawal from contact with the child. For the reason that both difficulties in forming a stable parental identity and parental burnout are significant predictors of difficulties experienced by parents such as depression and anxiety [32], it was predicted that they would have higher levels in parents who regret parenthood. Problems with forming parental identity and parental burnout are also positively correlated with a perfectionistic fear of evaluation [17, 33]. It was assumed that confirming the relationship between these characteristics (i.e. maladaptive perfectionism, parental identity, and parental burnout) and regretting parenthood would enable a better understanding of the dynamics of processes that lead to regretting having a child, and that this would constitute a significant supplement to Study 1.

The last issue covered in Study 2, which had not previously been the subject of scientific studies, was the relationship between regretting parenthood and the characteristics of the child. In the study presented here, the focus was on verifying whether the child had any special needs associated with his/her health (e.g. disability, chronic diseases). Earlier studies suggest that bringing up a child with special needs increases the risk of parental burnout, especially when it is particularly burdensome [34]. The study presented here sought to verify whether the parents of children with special needs are also in more danger of regretting that they have become parents at all.

### Materials and methods

#### Participants and procedure

The study was reviewed and approved by the Departmental Ethics Committee, SWPS University of Social Sciences and Humanities, Poznań, Poland (decision number 190110). The participants provided their written informed consent to participate in this study.

Study 2 was carried out using the same nationwide research panel as in Study 1. In the process of collecting the research sample, care was taken to ensure that the sample was as demographically diverse as possible, to represent the structure of the population in the best way. In order to verify the results of Study 1, which was the first study on this topic conducted in Poland, people who participated in Study 1 could not participate in Study 2 (this condition was ensured by the functionality of the research panel used, i.e. an invitation to participate in the study was not sent to people who participated in Study 1). The procedure used in Study 2 was the same as in Study 1. The participants were informed about the aim of the study, and gave their written consent to take part in it. The study was approved by the Departmental Ethics Committee of the author’s institution.

In Study 2, the participants were 1280 individuals between the ages of 18 and 30 (*M =* 26.11, *SD* = 2.99). The decision to lower the maximum age of the participants from that in Study 1 was dictated by a desire to focus on those parents who had just entered the parental role. The youngest parents constituted a minority in Study 1 (40% of the sample), which was another rationale behind this decision. The mean age of the participants’ children in Study 2 was 3.11 years (*SD* = 2.70); 63% of the participants had only one child, 31.3% had two children, and 5.6% had three or more. In the sample, 62.2% were women; 25.5% of the participants lived in villages, whereas the remainder inhabited towns and cities of different sizes; 53.9% were married, 38.2% were in informal relationships, and 7.9% were single. As regards education, 13.4% of the participants had primary, vocational or equivalent education, 39.9% had secondary education, and 46.6% were studying or had higher education; 38.8% said they had no financial difficulties, 55.7% claimed they only sometimes experienced financial problems, and 5.5% of the participants stated that their financial situation was bad. In comparison to the sample from Study 1, in Study 2 there were more individuals who had only one child, there were more students and individuals with higher education, and there were fewer participants who had the lowest level of education, fewer who claimed that their financial situation was bad, and fewer who were married. However, while the sample from Study 2 was not as representative as the sample from Study 1, it was still very diverse in terms of gender, social status, place of residence, education, etc.

#### Measures

*Regretting parenthood*. The manner of measuring regretting parenthood was analogous to that of Study 1.

*Maladaptive perfectionism*. Levels of perfectionistic fears of evaluation and rejection were measured with the use of two subscales from the Frost Multidimensional Perfectionism Scale (FMPS; [35], 1990; Polish adaptation [36]): Concern over Mistakes (9 items, e.g. *People will probably think less of me if I make a mistake*) and Doubt about Actions (4 items, e.g. *Even when I do something very carefully*, *I often feel that it is not quite done right*), which, after summing their results, creates an indicator of perfectionistic concerns [28]. This indicator was used in the study. Its reliability was .92. The other subscales of this questionnaire do not measure perfectionistic concerns and have been omitted.

*Parental identity*. The measurement of parental identity was conducted with the use of two questionnaires, which capture slightly different aspects of the characteristics of parental identity. The first questionnaire was the Utrecht-Management of Identity Commitments Scale: Parental Identity (U-MICS: PI, [17, 29]), which is a measure that is an adaptation of the scale developed by Crocetti, Rubini, and Meeus [37]. It enables the assessment of three dimensions of parental identity: Commitment (5 items, e.g. *Being a parent makes me feel sure of myself*; Cronbach’s alpha .90), which indicates the level of engagement in and identification with the role of a parent, In-depth exploration (5 items, e.g. *I try to find out a lot about my child/children*; Cronbach’s alpha .76), which measures the extent to which parents reflect on their current parental commitment and search for information on parenting and children, and Reconsideration of Commitment (3 items, e.g. *I often think that not having a child/children would have made my life more interesting*; Cronbach’s alpha .93), which measures the extent to which parents think that parenthood does not fit their needs and expectations (it is an indicator of the level of parental identity crisis). The second questionnaire was the Dimensions of Identity Development Scale (25 items, DIDS; [38]), in a version adapted for the measurement of parental identity [30], which was used to assess levels of the different identity processes distinguished in Luyckx et al.’s model of identity [38]. The process of developing the Polish adaptation was supervised by the author and was carried out using the back-translation method. The scale in its Polish adaptation preserves its intended, five-factor structure. It enables the measurement of five dimensions of parental identity: Exploration in breadth (5 items, e.g. *I think about different goals that I might pursue as a parent;* Cronbach’s alpha .86), which refers to the extent to which the parent considers and analyzes different ways of realizing the parental role, Commitment (5 items, e.g. *I have decided on the kind of parent I want to be;* Cronbach’s alpha .90), which pertains to the extent to which the person possesses a clear definition of her/his role as a parent, Exploration in depth (5 items, e.g. *I talk with my friends about the parenting of my child(ren);* Cronbach’s alpha .74), which is a process that refers to the extent to which the parent actively analyzes her/his commitment as a parent and searches for information about parenthood, Identification with commitment (5 items, e.g. *I feel certain about myself as a parent;* Cronbach’s alpha .89), which refers to the extent to which the parent identifies with the role of a parent and has a sense that it fits her/his expectations and needs, and Ruminative exploration (5 items, e.g. *I worry about what I am supposed to do as a parent;* Cronbach’s alpha .88), which is a dimension that points to difficulties in forming parental identity, manifesting themselves in sense of being lost, of uncertainty, and of a lack of goals in the realization of the parental role.

*Parental burnout*. The level of parental burnout was assessed with the use of the Parental Burnout Assessment (PBA) developed by Roskam, Brianda and Mikolajczak [39], in its Polish adaptation [40]. The questionnaire consists of 23 items that create four subscales: Exhaustion (9 items, e.g. *I feel completely run down by my role as a parent*), Contrast (6 items, e.g. *I don’t think I’m the good father/mother that I used to be to my child(ren)*), Feelings of being fed up (5 items, e.g. *I can’t stand my role as father/mother any more*), and Emotional distancing (3 items, e.g. *I do what I’m supposed to do for my child(ren)*, *but nothing more*). The sum of all the questionnaire items enables a general indicator to be obtained, which was used in the present study. The reliability of this indicator was .98.

*Having children with special needs*. The participants were asked the following question: *Does your child or any of your children have any chronic diseases or disabilities*? The participants could respond either *yes* or *no*. If the parent indicated that her/his child had chronic health problems, then she/he was asked: *Please evaluate to what extent this chronic disease or disability limits your child’s/children’s everyday functioning*. In this case, the parents would provide an answer on a five-point scale, ranging from 1-*to a very small extent*, *almost imperceptibly*, to 5-*to a great extent*, *the child is unable to do many things typical of its peers*.

#### Analytical strategy

In Study 2, an analogous analytical procedure to that of Study 1 was applied: the distribution of answers to the question about regretting parenthood was verified (H1), and differences between the parents who regretted and those who did not regret parenthood in respect of the analyzed demographic factors were checked using the *Chi-*Square test (H2), then differences between the parents who regretted and those who did not regret parenthood in respect of perfectionistic concerns, parental identity, and parental burnout were analyzed with the use of MANOVA (H3).

### Results

#### Descriptive statistics, mean differences, and correlations between quantitative variables

The descriptive statistics of the quantitative variables and mean differences are presented in Table 6. Men turned out to scored higher on perfectionistic concerns, difficulties with parental identity formation (higher reconsideration of commitment and ruminative exploration), and parental burnout. In turn, women obtained higher results on parental identity commitment, in-depth exploration, and exploration in breadth, suggesting that they identified with parenting more than men and were more prone to gather information about parenting and themselves as parents. Similarly, as in Study 1, marital status and financial situation turned out to be related to analyzed variables. Single parents were characterized by lower identity commitment and identification with commitment and were less prone to gather in-depth information about parenting. Similar effects were also related to financial difficulties; parents with substantial financial problems experienced higher parental burnout than those in a better financial condition. They were also less committed and identified with parenting and experienced higher difficulties forming a stable parental identity (higher reconsideration of commitment and ruminative exploration). The obtained results also pointed out significant relationships between study variables and having children with chronic illnesses or disabilities. Parents from this group experienced higher perfectionistic concerns and parental burnout. In terms of identity, they scored higher on reconsideration of commitment and ruminative exploration and were less committed, indicating their significant difficulties with forming a stable sense of parental identity.

**Table 6:** Descriptive statistics and mean differences in Study 2.

	Range	All sample (*N* = 1280)	Gender			Marital status				Financial situation				Child’s chronic illnesses or disabilities		
			Female	Male		Married	Informal	Single		No financial problems	minor financial problems	substantial financial problems		Yes	No	
			(*n* = 796)	(*n* = 484)		(*n* = 690)	(*n* = 489)	(*n* = 101)								
										(*n* = 497)	(*n* = 713)	(*n* = 70)				
		*M (SD)*	*M (SD)*	*M (SD)*	*F / p*	*M (SD)*	*M (SD)*	*M (SD)*	*F / p*	*M (SD)*	*M (SD)*	*M (SD)*	*F / p*	*M (SD)*	*M (SD)*	*F / p*
Perfectionistic concerns	1–5	2.87 (.83)	2.82 (.86)	2.96 (.78)	7.57***	2.87 (.84)	2.89 (.85)	2.82 (.77)	*ns*	2.83 (.82)	2.87 (.84)	3.18 (.82)	5.39**	3.06 (.85)	2.84 (.82)	11.98**
Commitment (U-MICS)	1–5	3.80 (.79)	3.87 (.77)	3.68 (.81)	17.45***	3.85^a^ (.80)	3.78^a^ (.76)	3.55^b^ (.86)	6.34**	3.88 (.82)	3.77 (.76)	3.61 (.89)	4.88**	3.68 (.79)	3.82 (.79)	4.83*
In-depth exploration (U-MICS)	1–5	3.99 (.62)	4.10 (.58)	3.81 (.66)	63.37***	4.00^a^ (.62)	4.01^a^ (.61)	3.83^b^ (.71)	3.23*	4.01 (.62)	3.98 (.63)	3.99 (.63)	*ns*	3.93 (.68)	4.00 (.61)	*ns*
Reconsideration of commitment (U-MICS)	1–5	2.06 (1.13)	1.89 (1.07)	2.33 (1.18)	47.86***	2.02 (1.15)	2.07 (1.11)	2.25 (1.13)	*ns*	2.01 (1.15)	2.06 (1.12)	2.36 (1.18)	3.03*	2.49 (1.18)	1.98 (1.11)	34.06***
Exploration in breadth (DIDS)	1–5	3.95 (.67)	4.04 (.65)	3.81 (.68)	35.15***	3.98 (.66)	3.93 (.65)	3.81 (.78)	*ns*	3.98 (.67)	3.93 (.67)	3.97 (.70)	*ns*	3.90 (.66)	3.96 (.67)	*ns*
Commitment (DIDS)	1–5	3.96 (.69)	4.05 (.67)	3.83 (.71)	31.55***	3.99^a^ (.69)	3.97^a^ (.67)	3.75^b^ (.81)	5.46**	4.06 (.68)	3.90 (.70)	3.92 (.69)	8.31***	3.86 (.72)	3.99 (.69)	5.42*
Exploration in depth (DIDS)	1–5	3.54 (.71)	3.56 (.71)	3.51 (.71)	*ns*	3.55 (.70)	3.56 (.71)	3.44 (.74)	*ns*	3.53 (.71)	3.55 (.71)	3.54 (.64)	*ns*	3.61 (.67)	3.53 (.71)	*ns*
Identification with commitment (DIDS)	1–5	3.87 (.72)	3.93 (.69)	3.77 (.75)	16.04***	3.90^a^ (.72)	3.87^a^ (.69)	3.68^a^ (.79)	4.06*	3.95 (.69)	3.83 (.72)	3.67 (.82)	7.03**	3.78 (.72)	3.89 (.72)	*ns*
Ruminative exploration (DIDS)	1–5	2.68 (.96)	2.57 (.96)	2.88 (.92)	32.20***	2.67 (.99)	2.69 (.93)	2.73 (.91)	*ns*	2.56 (.99)	2.73 (.93)	3.00 (.91)	9.19***	2.93 (.99)	2.64 (.95)	15.03***
Parental burnout	1–7	2.56 (1.43)	2.41 (1.33)	2.82 (1.54)	25.86	2.59 (1.46)	2.50 (1.37)	2.72 (1.45)	*ns*	2.33 (1.38)	2.66 (1.41)	3.22 (1.60)	16.13***	3.05 (1.55)	2.48 (1.39)	28.02***

* *p* < .05

** *p* < .01

*** *p* < .001, *ns*—not significant; post-hoc test Tukey HSD (groups with different index differed significantly).

Note: U-MICS: the Utrecht Management of Identity Commitments Scale; DIDS: the Dimensions of Identity Development Scale.

As regards correlation analysis (Table 7), in line with the predictions, it turned out that the parents with higher levels of perfectionistic concerns experienced difficulties in forming a stable sense of parental identity, had more doubts about whether parenthood fitted them, were characterized by greater confusion as to how to be a parent, and, finally, experienced greater parental burnout. These results remain in accord with earlier works on the relationship between perfectionism, identity, and parental burnout [17, 33, 41]. The parents who experienced a stronger parental identity crisis (high reconsideration of commitment and ruminative exploration) were also characterized by a higher level of parental burnout, which remains in accord with the data presented by Schrooyen, Beyers, and Soenens [30]. Age of the parent and number of children were only marginally correlated to study variables. However, it turned out that the difficulties experienced by the parents (higher ruminative exploration, reconsideration of commitment, parental burnout, low commitment) were positively associated with the children’s age. As children in the study sample were very young (mean age ca. 3 years), it may depict the process of deepening parental difficulties in the first years of parenthood in emerging adults. Higher levels of health issues/disability in children were also positively associated with parents’ difficulties such as high perfectionistic concerns, ruminative exploration, reconsideration of commitment, and parental burnout.

**Table 7:** Correlations between study variables in Study 2.

	2	3	4	5	6	7	8	9	10	11	12	13	14
1. Perfectionistic concerns	-.09**	.08**	.40***	-.01	-.11***	.24***	-.15***	.49***	.44***	-.03	-.06	.04	.30***
2. Commitment (U-MICS)	-	.56***	-.38***	.45***	.55***	.23***	.64***	-.21***	-.36***	.01	.06*	-.10	-.03
3. In-depth exploration (U-MICS)		-	-.29***	.57***	.57***	.43***	.52***	-.11***	-.28***	-.02	-.01	-.15***	.01
4. Reconsideration of commitment (U-MICS)			-	-.26***	-.30***	.06*	-.36***	.56***	.62***	.01	.01	.14***	.30***
5. Exploration in breadth (DIDS)				-	.69***	.48***	.60***	-.15***	-.26***	-.05	.01	-.10***	.01
6. Commitment (DIDS)					-	.37***	.71***	-.31***	-.37***	-.02	-.02	-.13***	-.09
7. Exploration in depth (DIDS)						-	.35***	.25***	.09**	-.01	-.01	-.05	.10
8. Identification with commitment (DIDS)							-	-.32***	-.43***	-.05	.02	-.10**	-.02
9. Ruminative exploration (DIDS)								-	.57***	-.02	.01	.08**	.28***
10. Parental burnout									-	.04	.08**	.12***	.33***
11. Age of the parent										-	.13***	.13***	-.06
12. Number of children											-	.26***	.05
13. Mean age of the children/the only child												-	.01
14. Health issues/disability level													-

* *p* < .05

** *p* < .01

*** *p* < .001.

Note: U-MICS: the Utrecht Management of Identity Commitments Scale; DIDS: the Dimensions of Identity Development Scale.

#### Regretting parenthood in the study sample

The evaluation of the answers to the question about regretting parenthood is presented in Table 8. It turns out that in the investigated sample, 10.7% of the emerging adult parents responded that, if they could make the decision once again, they would decide not to have children. This result is similar to that obtained in Study 1.

**Table 8:** Percentage of Polish parents aged 18–30 who regretted having a child (Study 2).

If you could travel back in time and once again make the decision, would you once again decide to become a parent?	No, I would choose a life without children	Yes, I would choose to have children
	*n* = 137	*n* = 1143
	10.7%	89.3%

#### Social and demographic factors in parents regretting parenthood

As in Study 1, no significant differences in respect of the frequency of regretting parenthood could be observed between women and men (Table 9); as in Study 1, it turned out that significant differences could be observed between parents with different marital statuses, *Chi*^2^ (2) = 20.91, *p* < .001, Cramer’s *V* = .13: among the individuals who were married, 8.1% regretted having children; among the individuals in informal relationships, the proportion was 11.9%; and among the single parents it was 22.8%. As in Study 1, differences could also be spotted between the individuals with different assessments of their financial situation, *Chi*^2^ (2) = 11.71, *p* < .01, Cramer’s *V* = .10: among the participants who claimed that they did not have financial difficulties, 9.5% regretted parenthood, among the individuals with minor financial difficulties the figure was 10.4%, and among the parents with serious financial problems, the percentage who regretted having children was 22.9%. Also, a relationship could be observed–not too strong, although significant–between having a child with special needs and regretting parenthood, *Chi*^2^ (1) = 5.20, *p* < .05, Cramer’s *V* = .06. Among the parents who had at least one child with special health needs, the percentage of those who regretted parenthood was 15.7%, whereas, among the parents whose children had no such health issues, this percentage was 9.8%. Among the individuals bringing up a child with special needs, no relationship between regretting parenthood and the level of the child’s health problems could be observed, *F* (1,252) = 3.18, *p* = .076, eta^2^ = .01.

**Table 9:** Regretting parenthood and gender, marital status, and financial situation (Study 2).

	If you could travel back in time and once again make the decision, would you once again decide to become a parent?		
	No, I would choose a life without children	Yes, I would choose to have children	*Chi*^2^
			*p*
	*n* = 160	*n* = 1015	
Gender			
Female (*n* = 796)	9.8%	90.2%	*ns*
Male (*n* = 484)	12.2%	87.8%	
Marital status			
Married (*n* = 690)	8.1%	91.9%	*X*^2^ = 20.91
Informal relationships (*n* = 489)	11.9%	88.1%	*p* < .001
Single (*n* = 101)	22.8%	77.2%	Cramer’s *V* = .13
Financial situation			
No financial problems (*n* = 497)	9.5%	90.5%	*X*^2^ = 7.08
Minor problems (*n* = 713)	10.4%	89.6%	*p* < .05
Bad financial situation (*n* = 70)	22.9%	77.1%	Cramer’s *V* = .08
Does your child/any of your children have any chronic illnesses or disabilities?			*X*^2^ = 6.17
			*p* < .05
Yes (*n* = 197)	15.7%	84.3%	Cramer’s *V* = .07
No (*n* = 1083)	9.8%	90.2%	

Additionally, there was no relationship between regretting parenthood and place of residence (a village or a city of some size). Unlike in Study 1, differences were detected among participants with different education levels, *Chi*^2^ (2) = 10.84, *p* < .01, Cramer’s *V* = .09: the smallest percentage of parents regretting parenthood could be observed in the case of participants with higher education or who were still studying (8%), a slightly higher percentage was observed among the participants with secondary education (11.9%), and the highest percentage of parents who regretted having children could be seen among the individuals with primary, vocational or equivalent education level (16.3%).

#### Psychological functioning and health in parents regretting parenthood

Table 10 presents the differences between the two groups of parents in respect of perfectionistic concerns, parental identity, and parental burnout. The MANOVA revealed a significant multivariate effect, Wilks’ *λ* = .83, *F* (10, 1269) = 25.66, *p* < .001, *η*^2^ = .17. The effect size was twice as high as in Study 1. This was the result, in the first place, of considerable differences between the two groups in respect of the sense of parental identity and parental burnout. The parents who regretted having children were characterized by stronger perfectionistic concerns, lower parental identity commitment, and also lower activity in the exploration of the parental domain, both when it came to considering different ways of realizing the role of a parent (exploration in breadth) and in relation to searching for detailed information about parenthood and children (in-depth exploration). In this group, we could also observe strong parental identity crisis, manifesting itself in low identification with the role of a parent (reconsideration of commitment) and uncertainty about how to fulfill this role (ruminative exploration). Finally, the parents who regretted having children experienced markedly higher levels of parental burnout. The greatest effect size could be observed in the dimensions of commitment and reconsideration of commitment (U-MICS), identification with commitment (DIDS), and parental burnout.

**Table 10:** Mean differences between the parents regretting and not regretting parenthood in Study 2.

	No, I would choose a life without children	Yes, I would choose to have children		
	*M (SD)*	*M (SD)*	*F*	eta^2^
Perfectionistic concerns	3.10 (.88)	2.85 (.83)	10.76**	.01
Commitment (U-MICS)	3.12 (.99)	3.88 (.72)	125.19***	.09
In-depth exploration (U-MICS)	3.67 (.72)	4.03 (.60)	40.50***	.03
Reconsideration of commitment (U-MICS)	3.17 (1.10)	1.92 (1.06)	167.91***	.12
Exploration in breadth (DIDS)	3.72 (.82)	3.98 (.64)	18.05***	.01
Commitment (DIDS)	3.61 (.83)	4.01 (.66)	42.06***	.03
Exploration in depth (DIDS)	3.48 (.80)	3.55 (.70)	1.49	< .01
Identification with commitment (DIDS)	3.34 (.80)	3.94 (.68)	91.20***	.07
Ruminative exploration (DIDS)	3.16 (.85)	2.62 (.96)	38.15***	.03
Parental burnout	3.77 (1.57)	2.41 (1.33)	120.35***	.09

* *p* < .05

** *p* < .01

*** *p* < .001.

Note: U-MICS: the Utrecht Management of Identity Commitments Scale; DIDS: the Dimensions of Identity Development Scale.

### Discussion

As a result of a decrease in fertility in developed countries, governments have started to take various actions to increase the number of children born. The situation is no different in Poland, where the fertility rate is one of the lowest among the EU countries [42]. However, when pursuing the aim of increasing the number of children born, we often forget that parenthood is a difficult and stressful task, and that young people, who are those who most frequently become parents, sometimes fail to cope with this new reality successfully. In order to plan support for young parents skillfully, both at the level of national policy and in relation to individual support provided by specialists, it is necessary to conduct an initial diagnosis of the number of parents for whom the realization of the parental role poses serious problems. Among the difficulties that can be experienced by a person who has decided to have a child, one of the most serious is to arrive at the conclusion that it was a bad decision. Because one cannot withdraw from parenthood at one’s own request, and because regretting parenthood can lead to mental problems and negative attitudes towards children [18], this should be an area of particular interest for social researchers, including psychologists. Unfortunately, our knowledge about the prevalence of regretting parenthood and about its causes has, so far, been very limited. The studies presented here, based upon the analysis of two large Polish samples, one of which was a representative sample, aimed to fill this gap.

Earlier survey studies, conducted on an American [1] and a German [12] sample, estimated that the percentage of parents who claimed that if they could make a choice again they would not decide to have children was between 7 and 8%. However, the results obtained in Study 1 and Study 2 indicate that this percentage may be higher in Poland, perhaps even 50% higher. As a result, the predictions formulated in Hypothesis 1 can be considered to be only partially confirmed. In Study 2, the percentage of parents who regretted having children was slightly lower than in Study 1 (10.7% and 13.6%, respectively), but it is the result of Study 1 that needs to be treated as more trustworthy, because the first research sample was more representative; in the second sample, there was a slight overrepresentation of well-educated individuals and of those with a healthy financial situation, which could result in a lower percentage of people who regretted parenthood. The results obtained point to two issues. First of all, it seems that, regardless of the country in which the study is conducted, one can expect to find parents who think that having a child was a bad decision. Additionally, it turned out once again that the number of such people is so large that neither the individuals who shape social policy in a given country nor researchers can fail to recognize this issue. Secondly, in the Polish population, the percentage of people who regret parenthood is higher than in the other populations examined so far. The research methodology applied was very similar to that used in the German study, so that it seems that the measurement method could not have been responsible for these differences. Unlike Germany, Poland has one of the strictest anti-abortion regimes in Europe. Abortion can only be performed when the mother’s health or life are endangered, when a serious defect/disease in the foetus threatens its life or the life or health of the mother, or when the pregnancy is the result of crime. Consequently, in Germany there are over 100,000 abortions performed each year, whereas in Poland the number is around 1000 [43]. This may mean that in Poland, the number of people who become parents and who are not convinced that this decision is the right one is higher because of the lack of any other option. Another factor that distinguishes Poland from the other two countries mentioned earlier is the level of economic development. Both the German study and the studies presented in this article indicate that one of the factors that increases the probability of regretting parenthood is the poor financial situation of the family. Poland, despite making a gigantic leap over the last thirty years since the Communist dictatorship was overthrown, is still a country in which incomes (even when purchasing power parity is taken into account) are considerably lower than in Germany or in the US, which can result in a higher percentage of parents for whom having a child is too great a financial burden; this may have had an effect on the results obtained. In Study 1 only 32% of the parents surveyed said that they had no financial problems at all; in Study 2 it was 39%, which may indicate the scale of the problem. The obtained results are also in accordance with the recent studies suggesting that Polish parents are among the most at risk of parental burnout in Europe [40] which lead to the conclusion that further intercultural studies on regretting parenthood, conducted on national samples, need to be conducted, so that the factors indicated here (i.e. abortion law and economy) can be fully verified.

In the study, there were two demographic factors that seemed to lead to the greatest increase in the risk of regretting parenthood. The first was the aforementioned poor financial situation of the parents, while the second one was being a single parent. In the case of single parents (which pertains to both women and men), their evaluation of parenthood as something that should not have happened can result from the fact that some of them, after splitting up with the other parent, cannot count on sufficient support. We should also remember that one of the most frequent sources of regret among adults are decisions associated with romantic relationships, which has been observed by Dijkstra and Barelds [7]. The results of the present study suggest that regretting decisions from the romantic domain may also translate into a negative evaluation of becoming a parent, and that sometimes a parent starts to regret not only that s/he entered into a relationship with a given person, but also that s/he had a child with this individual.

Interesting, and in places even surprising, results were obtained concerning gender. Among women and men, the percentage of parents regretting having children was similar, which is in line with research conducted in Germany [12]. On the other hand, many differences were observed between men and women in the samples studied. Women experienced more depressive symptoms, anxiety, vegetative symptoms, but were also characterized by a more stable sense of parental identity. On the other hand, men were characterized by more intense traumatic experiences in childhood and stronger parental burnout and parental identity crisis. Thus, although the situation of women-mothers and men-fathers differs, this does not translate into visible differences in terms of their greater or lesser propensity to regret parenthood. This observation may imply that regretting parenthood among mothers and fathers has different causes, which is a very promising area for future research.

In terms of gender, the finding of stronger parental burnout among fathers in Study 2 is surprising. Previous research on parental burnout suggests that women experience this syndrome to a greater extent [25, 44], which was also observed in research previously conducted in Poland [40]. What distinguishes the parents who took part in Study 2 from other studies on parental burnout is their young age (women *M* = 26.00, men *M* = 26.30). In earlier studies, the samples of parents were usually older by about ten years. Therefore, it is reasonable to consider the possibility that among the youngest parents, in the period of emerging adulthood (aged 18–30), they may be the fathers who experience stronger parental burnout. This is an issue worth further analysis in the future.

Regardless of the fact that these particular demographic factors turned out to be connected with regretting parenthood, which remains in accord with the predictions formulated in Hypothesis 2, we would not be justified in claiming that regretting parenthood is typical of individuals in a poor financial situation or of divorced people. Despite the fact that a slightly higher percentage of parents who regretted parenthood could also be observed among the parents who had children with special needs, this difference was only 5%, which it is difficult to consider as a strong effect. Conversely, the results obtained clearly indicate that parents who regret parenthood come from all layers of society: they are present among the wealthy and the well-educated as well among the poor and those with only primary education, some of them have children with special needs and some of them do not, and some of them are married or in informal relationships and some of them do not have a partner. In line with the predictions included in Hypothesis 3, it seems that, to be able to obtain a good understanding of a parent who regrets having a child, it is necessary to refer to factors at the psychological level [see also 45].

The results of Study 1 justify a search in childhood for the conditionings of regretting parenthood. It turns out that parents who regret having children were more often raised in an environment characterized by violence and rejection. Such conditions in childhood lead to changes in the functioning of the hypothalamic-pituitary-adrenal (HPA) axis, increasing the frequency and intensity of stress experiences, and contributing to impairments in cognitive functions that underlie self-regulation and, in consequence, to psychopathology [46]. This accords with the results of Study 1, as we could observe that the parents who regretted having children also manifested more symptoms associated with anxiety or depression, or somatic symptoms. Transition to parenthood and raising children is a great burden for almost all parents, and successful adaptation to this role requires many resources [47]. In the case of individuals who have behind them a childhood marked by traumatic experiences and problems with mental and somatic health, the task of being a parent can be too burdensome, can offer few rewards, and can yield no satisfaction [48], and, as demonstrated by the results obtained, this can also lead to regretting the decision to become a parent.

In accord with the observations about adverse childhood experiences and mental health (Study 1) are the observations pertaining to differences between the compared groups in respect of perfectionistic concerns (Study 2), which makes it possible to bind the two studies together theoretically. Strong fear of external evaluation and of not meeting expectations, which turned out to be higher among the parents who regretted having a child, are usually also a consequence of a dysfunctional rearing environment [35, 49], and perfectionistic concerns themselves are an important predictor of psychopathology. The fact that parents who regret parenthood have greater sensibility to external evaluation accords with the observations made by Donath [14] that one of the factors that stands behind this phenomenon can be the social and cultural pressure to have children. Individuals who find it more difficult to resist this pressure can more often make the decision to become a parent under the influence of social norms and not personal needs, which can increase the risk of future regrets about making this decision.

The moment of becoming a parent marks the beginning of one of the most important processes in adulthood, namely forming a stable parental identity [29]. The development of parental identity is based on comparing one’s own experiences in the realization of the parental role with one’s expectations, needs, and values. A satisfactory course through this process leads to identification with the decisions one has made and to defining oneself through the prism of these decisions. In Study 2, it was observed, however, that the individuals who regretted having children experienced strong and marked parental identity diffusion. They identified with the role of a parent only to a low extent, and they also had a much weaker motivation to consider issues connected with parenthood and to search for deeper information about it. In this group of parents, we could observe doubts about how to realize the parental role and a conviction that parenthood does not fit them. Piotrowski [17] suggested that regretting parenthood can be the final effect of disturbances in the development of parental identity, although he did not provide conclusive proof of that. Nevertheless, the results of Study 2 seem to corroborate that this may be possible. In turn, Schrooyen, Beyers and Soenens [30] have suggested that disturbances in parental identity lead to parental burnout. This thesis has also been confirmed by the results we obtained. On the basis of the theoretical assumptions presented by Piotrowski [29] and Schrooyen, Beyers and Soenens [30], we can presume that difficulties in forming a stable parental identity after entering the parental role can be an introduction to parental burnout which, in turn, can lead to regrets about ever becoming a parent which makes it important to further study the relationships between these different forms of disturbances in parenting. There are many studies that have shown that chronic difficulties in forming a stable sense of identity are closely related to mental health issues [50], and that a sense of parental identity is no exception here [32]. Therefore, it seems that the situation of parents who regret having children is characterized by many difficulties of both a psychological and a social nature, and this needs to be taken into consideration in the process of developing preventive and supportive actions.

#### Limitations and recommendations for future studies

Despite yielding a great deal of new information about the situation of parents who regret parenthood, the results obtained need to be looked at in the light of certain limitations of the research that was conducted. First of all, the method applied to measure regretting parenthood was relatively simple, and it did not provide an opportunity to capture differences in the level of this phenomenon. Using a more complete picture of parental regret is recommended in the future studies. The parents in the investigation had to take a stance on one of the two sides of the question, and could not specify to what extent they regretted parenthood, which, as demonstrated by the survey conducted in Germany [12], could be a source of interesting data. Secondly, the conclusions formulated in the present article are based on the cross-sectional approach and on a single measurement. Thus, they do not take into account changes that may appear in a parent’s perception of parenthood. In the future, it would be advisable to conduct a longitudinal study in order to evaluate the extent to which regretting parenthood is stable over time. The longitudinal design would also provide useful information about the direction of causality between mental health issues, parental identity, parental burnout and parental regrets. Thirdly, only self-description methods, pertaining solely to the parent her-/himself, were applied in the two studies. In future studies, it would be worth considering different sources of information about the parent (e.g. information provided by the person’s partner) to verify whether the person’s subjective sense of regretting parenthood is also connected with this person being perceived differently by other people. Fourthly, the study focused on the situation of the parent her-/himself, failing to include the relationship with the child. Since it has been demonstrated by East, Chien, and Barber [18] that regretting parenthood is associated with harsh parenting, in future studies a deeper analysis of how regretting parenthood influences the development of a child should be conducted. An analysis should also be undertaken into how the national regulations on abortion availability influence the percentage of parents who regret becoming parents, which requires broad, multicultural comparisons.

Among the limitations of the conducted research, the cause-and-effect relationships require particular commitment from the researchers. The relationships between perfectionism, identity, burnout, and parenting regret described above are based mainly on theoretical assumptions [17, 28, 30, 50]. As yet, there have been no longitudinal studies published of perfectionism and parental burnout and sense of parental identity, nor are there longitudinal data on parenting regret and the psychological characteristics examined in this study. Therefore, any assertions about potential causal relationships should be treated as hypotheses for future longitudinal studies. Increasing regret resulting from having children may affect parent functioning [18], and it is not precluded that it might also lead to an increase in parental burnout, identity crisis, and perfectionist concerns. Another area requiring in-depth longitudinal research is the relationship between childhood traumatic experiences and regretting parenthood. From the research presented here, we can only infer a correlation between these experiences, but the mechanism behind this link is still unknown. Given the limited research interest to date in the topic of regretting parenthood, it appears that this area of research may yet provide many breakthroughs.

## Conclusions

The role of a parent is one of the most important areas of activity for adult individuals [40]. Studies have shown that, although for the majority of parents parenthood is a source of satisfaction and development, there are parents for whom it can be the cause of stress, and even of mental problems. The objective of the research presented here was to learn more about the very group of parents for whom parenthood has become a burden. We sought an answer to the question of the frequency of occurrence in the population of the extreme form of low adaptation to parenthood that manifests itself in regretting undertaking this role. The study showed that, in the Polish population, this frequency may be as much as 13% of parents between emerging and middle adulthood, that is, one in eight parents. On the scale of a country the size of Poland, with a population of approximately 38 million, this can translate into several millions of parents, and on the scale of the European Union (approximately 440 million) or North America (approximately 580 million), into tens of millions more. Not only does regretting parenthood constitute a burden for the parent her-/himself, but it also disrupts the realization of the role of caregiver and educator, and, in all likelihood, it also has a negative influence on the relationship with the partner, on the person’s health, and maybe on the relationship with the child. Thus, it is a phenomenon that affects the entire family and the wider society, and intensified actions aiming at understanding and contradicting it are required from the scientific community. Taking into consideration the estimated number of people who regret parenthood, this phenomenon cannot be ignored, either, by those individuals responsible for shaping national policies, who should advocate that the decision about becoming a parent should be a conscious process. For individuals who regret having children, a support network should also be created, connecting, among others, physicians, psychiatrists and psychologists, in order to be able to provide them with professional help in this crisis.
