# Correction: How many parents regret having children and how it is linked to their personality and health: Two studies with national samples in Poland

**DOI:** 10.1371/journal.pone.0314900

**Published:** 2024-12-02

**Authors:** The PLOS One Staff

In Abstract section, there is an error in the first sentence. The correct sentence is: Surveys conducted over the last few years on representative samples in the US and Germany suggest that the percentage of parents who regret having children is approximately 7–8%.

The publisher apologizes for the error.

## References

[1] Piotrowski K (2021) How many parents regret having children and how it is linked to their personality and health: Two studies with national samples in Poland. PLoS ONE 16(7): e0254163. 10.1371/journal.pone.0254163 34288933 PMC8294566

